# High-Performance Acoustic Transducers with Exfoliated NbSe_2_ Nanosheets and Hybrid Force Mechanisms

**DOI:** 10.3390/ma18040763

**Published:** 2025-02-09

**Authors:** Dong-Kwan Lee, Won-Jin Kim, Kun-Woo Nam, Sung-Hoon Park

**Affiliations:** Department of Mechanical Engineering, Soongsil University, 369 Sangdo-ro, Dongjak-Gu, Seoul 06978, Republic of Korea; kd2890@naver.com (D.-K.L.); dnjswlso@naver.com (W.-J.K.); kwn1522@naver.com (K.-W.N.)

**Keywords:** TMDCs, NbSe_2_, exfoliation, acoustic transducers, sound pressure level

## Abstract

The transition metal dichalcogenide (TMDC) NbSe_2_ is a highly conductive and superconducting material with great potential for next-generation electronic and optoelectronic devices. However, its bulk form suffers from reduced charge density and conductivity due to interlayer van der Waals interactions. To address this, we exfoliated NbSe₂ into nanosheets using lithium-ion intercalation and utilized them as diaphragms in acoustic transducers. Conventional electromagnetic and electrostatic mechanisms have limitations in sound pressure level (SPL) performance at high and low frequencies, respectively. To overcome this, we developed a hybrid force mechanism combining the strengths of both approaches. The NbSe₂ nanosheets were successfully prepared and analyzed, and the NbSe_2_-based hybrid acoustic transducer (N-HAT) demonstrated significantly improved SPL performance across a wide frequency range. This study offers a novel approach for designing high-performance acoustic devices by harnessing the unique properties of NbSe_2_.

## 1. Introduction

2D materials such as graphene have been extensively studied, with numerous research works published due to their outstanding physical properties [1,2,3,4]. Among these, Transition-metal dichalcogenides (TMDCs) are a versatile class of 2D materials with applications in diverse fields such as biosensors, solar cells, batteries, and transistors [5,6,7,8,9,10,11,12,13]. TMDCs have a unique MX_2_ stoichiometry, where a transition metal (M) is covalently bonded to chalcogenides (X), forming a layered structure governed by van der Waals forces [14,15,16,17,18,19]. This layered structure enables properties such as superconductivity, semiconducting behavior, and ferromagnetism, depending on the material composition [12,13,14,15,16,20,21,22,23,24]. Among TMDCs, MoSe_2_ and MoS_2_ exhibit wide bandgaps and semiconducting properties [25,26,27], while NbSe_2_ is notable for its exceptional electrical conductivity (~10^6^ S/m) at room temperature and its superconducting properties below ~7.2 K [28,29]. However, the bulk form of NbSe_2_ suffers from reduced conductivity due to interlayer interactions, necessitating its exfoliation into nanosheets to unlock its full potential [30,31]. Therefore, for effective utilization of NbSe_2_ in various electronic applications, exfoliating the bulk crystal into thin 2D nanosheets with a thickness of just a few nanometers is a critical challenge in terms of both economic feasibility and performance enhancement.

**Figure 1:**
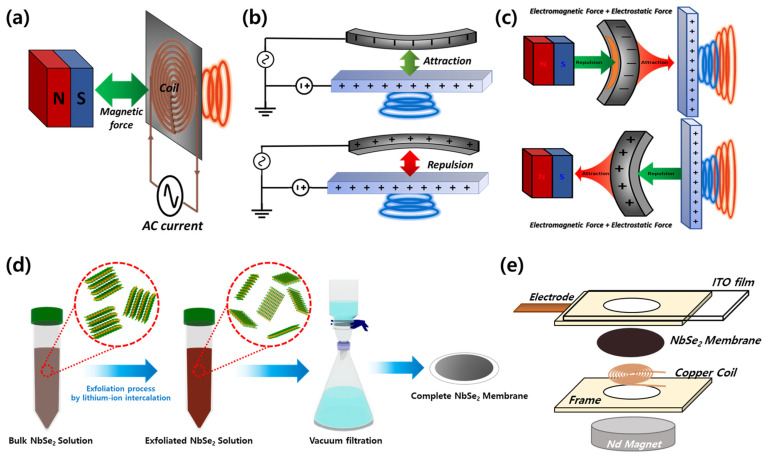
Schematic illustrations of the mechanisms for (**a**) electromagnetic, (**b**) electrostatic, and (**c**) hybrid acoustic transducers; (**d**) the lithium-ion intercalation exfoliation process and subsequent filtration; (**e**) the structural design of the N-HAT.

Since their invention, acoustic transducers have become essential components in modern electronic devices such as televisions, automobiles, and smartphones [32,33,34]. These devices convert electrical signals into sound waves using electromagnetic or electrostatic mechanisms, the most widely adopted methods in commercial applications [35,36,37]. The electromagnetic mechanism uses a coil attached to a diaphragm, where an alternating current (AC) generates a magnetic field that interacts with a permanent magnet to produce vibrations and sound waves, as depicted in Figure 1a. However, this method suffers from reduced sound pressure level (SPL) performance at high frequencies due to increased impedance and non-uniform force distribution [38]. Conversely, as shown in Figure 1b, the electrostatic mechanism relies on Coulomb forces between a conductive diaphragm and a charged electrode plate. While this method provides superior high-frequency performance, it exhibits reduced SPL at low frequencies due to limited diaphragm displacement [39,40,41]. These contrasting limitations lead to uneven frequency responses, highlighting the need for innovative designs to overcome these challenges [42,43].

Numerous studies have explored the applications of NbSe_2_ in various electronic fields. Liu et al. investigated the structural and thermal spin transport properties of NbSe_2_, revealing novel spin-related behaviors under strain-free conditions and demonstrating its potential for next-generation spintronic devices [44]. Yang et al. highlighted the nonlinear optical properties of 2D NbSe_2_ nanosheets and their use in ultrafast photonics. Their research confirmed the feasibility of liquid-phase exfoliated NbSe_2_ in advanced photonic devices, emphasizing its high optical responsiveness [45]. Zhu et al. examined the thermoelectric properties of few-layer NbSe_2_, focusing on its potential for energy-efficient applications. They found a strong correlation between the material’s electronic structure and thermoelectric performance, paving the way for its integration into sustainable energy devices [30]. Despite these advancements, no study has yet reported on the exfoliation of high-electrical-conductivity NbSe_2_ nanosheets from bulk NbSe_2_, their fabrication into acoustic transducer diaphragms, or their evaluation in various acoustic transducer mechanisms.

In this study, a hybrid force mechanism was introduced, as shown in Figure 1c, to address the limitations of electrostatic mechanisms in low-frequency ranges and electromagnetic mechanisms in high-frequency ranges. This mechanism combines both forces to act simultaneously on the diaphragm, leveraging their complementary strengths to significantly enhance SPL performance across a broader frequency range. Bulk NbSe_2_ was exfoliated into nanosheets using a lithium-ion intercalation method, enabling the fabrication of diaphragms with excellent electrical conductivity and mechanical stability. NbSe_2_ was chosen for its outstanding electrical conductivity (~10^6^ S/m) and robust mechanical properties, which ensure reliable performance under dynamic conditions [24,25]. These features make NbSe_2_ an ideal material for integrating with the hybrid mechanism, offering a unique combination of properties that surpass alternative materials like graphene or MoS_2_ in this application [26,27]. The exfoliation process was thoroughly evaluated using scanning electron microscopy (SEM), Raman spectroscopy, and atomic force microscopy (AFM), confirming successful exfoliation. An acoustic transducer system capable of selectively operating under electrostatic, electromagnetic, or hybrid force mechanisms was constructed using NbSe_2_ nanosheet diaphragms. Frequency response measurements demonstrated that the hybrid force mechanism achieved enhanced SPL performance across a wide frequency range. This study presents the design and performance evaluation of the NbSe_2_-based hybrid force acoustic transducer (N-HAT), leveraging exfoliated NbSe_2_ nanosheets and the hybrid force mechanism. The results highlight the feasibility and performance advantages of N-HAT, offering a novel approach for advanced acoustic device applications.

## 2. Materials and Methods

### 2.1. Exfoliation of Bulk State NbSe_2_

First, 10 mg of NbSe_2_ powder (>99.8%, Alfa Aesar, Haverhill, MA, USA) was placed into a 30 mL vial. To ensure a safe environment for lithium-ion intercalation, the gas inside the vial was replaced with nitrogen. Once the vial atmosphere was sufficiently purged with nitrogen, 5 mL of n-butyllithium solution (1.6 M, Sigma Aldrich, St. Louis, MO, USA) was injected into the vial. The vial was then subjected to bath sonication at 60 °C for 90 min to facilitate lithium-ion intercalation between the layers of NbSe_2_. Next, hexane was added to the solution to remove unreacted residues. Following this, DI water was introduced, and a 5 min bath sonication was performed to initiate the exfoliation process. During this process, the lithium ions intercalated between the NbSe_2_ layers react with water, releasing hydrogen gas. The release of hydrogen gas creates mechanical forces that exfoliate the NbSe_2_ nanosheets.

As shown in Figure 1d, the exfoliated NbSe_2_ nanosheets were efficiently collected by transferring the solution into conical tubes and performing centrifugation at 600 rpm for 20 min to isolate the supernatant. To further enhance the exfoliation yield, an additional 5 min sonication was conducted. The resulting solution was then subjected to vacuum filtration through a PES filter membrane, allowing the solvent to pass through while the NbSe_2_ nanosheets were deposited onto the membrane. Finally, the membrane was dried in a 70 °C oven to remove residual solvents, yielding a complete NbSe_2_ membrane ready for further use.

### 2.2. Fabrication of NbSe_2_-Hybrid Acoustic Transducer

To fabricate the N-HAT, a supporting frame was designed and 3D printed using PLA filament. The supporting frames were crafted into square donut-shaped structures with outer dimensions of 30 × 30 mm and a central circular opening with a 20 mm diameter. The thickness of the frames was kept at 1 mm to ensure sufficient interaction between the NbSe_2_ membrane and the electrode plate without physical contact. A planar copper coil with 16 turns was then prepared and securely attached to the underside of the NbSe_2_ membrane using an insulating adhesive film to ensure durability. The membrane assembly was positioned between two supporting frames. Before assembly, a copper electrode for electrostatic force was affixed to the supporting frame. During assembly, the NbSe_2_ membrane was carefully and firmly sandwiched between the two frames to ensure complete and consistent contact. Next, a transparent and electrically conductive Indium-Tin Oxide (ITO) film was attached to the side of the supporting frame facing the NbSe_2_ membrane to facilitate electrostatic interaction. On the opposite side, a permanent magnet was attached to the supporting frame facing the copper coil to enable electromagnetic interaction. This completed the assembly of the fully functional N-HAT device. The structure was carefully designed to allow for the integration of both electrostatic and electromagnetic mechanisms, leveraging the hybrid force approach for improved acoustic performance across a wide frequency range.

### 2.3. Characterization

To evaluate the exfoliation state of NbSe_2_, SEM (Gemini SEM 300, ZEISS, Oberkochen, Germany) imaging was performed. For this, the NbSe_2_ dispersion obtained from the exfoliation process was spin-coated onto a silicon wafer, and bulk-state NbSe_2_ powder was also imaged for comparison. Additionally, AFM (NX-10, Park Systems, Suwon, Republic of Korea) analysis was conducted to assess the morphology of the exfoliated NbSe_2_ and measure its thickness. Furthermore, Raman spectroscopy (LabRam Aramis, Horiba Jobin Yvon, Kyoto, Japan) was used to analyze peak changes before and after exfoliation. These evaluations confirmed that NbSe_2_ was successfully and efficiently exfoliated. After verifying the morphology and structure, the electrical conductivity of the exfoliated NbSe_2_ was confirmed using a four-point surface resistivity meter (RC2175, EDTM, Toledo, OH, USA). In this study, all experiments were conducted under strictly controlled conditions of 25 °C and 15% relative humidity using a dehumidifier and temperature control system, ensuring that the performance of the NbSe₂-based system was not affected by environmental factors.

For the sound pressure level (SPL) test of the fabricated N-HAT, a function generator (~20 V_pp_, DG952, RIGOL Technologies Co., Suzhou, China) was used. Channel 1 of the generator was connected to the planar copper coil using a BNC cable, while Channel 2 was connected to the input of an amplifier (TA 100-050M, RIGOL Technologies Co., Suzhou, China), which could amplify the AC signal to a maximum of 180 V_pp_. The amplifier output was connected via a BNC cable to the copper electrode on the NbSe_2_ membrane and the ground. Additionally, to polarize the ITO film electrostatically, a power supply was connected to apply a direct current (DC) voltage. The complete N-HAT setup was placed inside a soundproof chamber, and a calibrated microphone (UMIK-1, miniDSP, Hong Kong) was used to measure the SPL. Based on the SPL measurements, frequency response data of the N-HAT were obtained, enabling an evaluation of its acoustic performance across a wide frequency range.

## 3. Results and Discussion

### 3.1. Morphological Analysis of Exfoliated NbSe_2_

The NbSe_2_ nanosheet features a sandwich-like structure, where two selenium (Se) layers strongly bonded with covalent interactions encapsulate a single niobium (Nb) layer above and below. Typically, synthesized NbSe_2_ exists in a bulk crystalline state with a layered structure, wherein the NbSe_2_ nanosheets are held together by van der Waals forces. Considering the nanosheet’s X–Y plane, the van der Waals interactions align along the Z-axis, binding the layers together. However, van der Waals force is significantly weaker compared to the covalent bonds within the X–Y plane, making it possible to exfoliate bulk NbSe_2_ into mono- or few-layer nanosheets through various methods.

**Figure 2:**
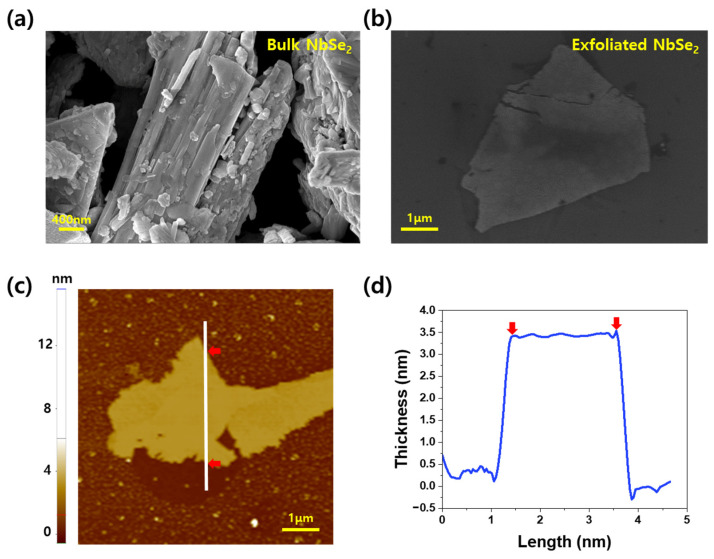
SEM images of (**a**) bulk-state NbSe_2_ and (**b**) exfoliated NbSe_2_; (**c**) AFM image of exfoliated NbSe_2_ and (**d**) thickness profile of the NbSe_2_ nanosheet (Red arrows mark the start and end of exfoliated NbSe₂ in the AFM image and graph).

The lithium-ion intercalation method efficiently exfoliates NbSe_2_ by leveraging the interaction between lithium ions and water molecules. During the exfoliation process, lithium ions are inserted between the layers of bulk NbSe_2_ in a sonication bath. Subsequently, the addition of DI water triggers interactions between the intercalated lithium ions and water molecules, releasing hydrogen gas. The released hydrogen gas overcomes the van der Waals interactions along the Z-axis, enabling the exfoliation of nanosheets from the bulk structure. In Figure 2a, SEM imaging of bulk NbSe_2_ crystal is presented. As shown, bulk NbSe_2_ exhibits a 3D particulate form rather than the thin, 2D sheet-like morphology characteristic of exfoliated nanosheets. Conversely, Figure 2b shows SEM images of exfoliated NbSe_2_, spin-coated onto a Si wafer. Unlike bulk NbSe_2_, the exfoliated material displays the expected thin, 2D nanosheet morphology.

Further analysis using AFM, as shown in Figure 2c,d, evaluated the thickness of the exfoliated NbSe_2_ nanosheets. The X–Y plane of the nanosheets spans several micrometers, while the thickness is approximately 3.5 nm, confirming the successful exfoliation of the material into a true nanosheet structure.

### 3.2. Raman Spectra Analysis of NbSe_2_

After conducting morphological analysis of NbSe_2_’s exfoliation from bulk state to nanosheets using SEM and AFM, Raman spectroscopy was employed to analyze changes in the crystal structure. Raman spectroscopy is a technique that investigates molecular vibrations, rotations, and other low-energy modes by examining the interaction between light and matter. When a laser beam is directed onto a sample, most photons undergo Rayleigh scattering (retaining their original energy), but a small fraction interact with molecular vibrations or lattice modes, resulting in energy shifts known as Raman scattering. These shifts manifest as Stokes (lower frequency) or anti-Stokes (higher frequency) shifts.

Raman signals contain information about the intrinsic vibrational modes of the material, providing insights into the crystal structure, layer number, defects, and electron-lattice interactions. For exfoliated NbSe_2_, changes in the position and intensity of Raman peaks correlate with the number of layers, offering a means to study interlayer interactions and crystal symmetry. Additionally, Raman analysis compares structural changes between bulk NbSe_2_ and exfoliated nanosheets, detecting potential defects or asymmetry induced during the exfoliation process.

Raman spectra measurements revealed distinct differences between bulk NbSe_2_ and exfoliated NbSe_2_. When laser light interacts with NbSe_2_, two characteristic peaks are observed: the A_1g_ peak, associated with atomic vibrations along the Z-axis, and the E^1^_2g_ peak, related to in-plane vibrational modes in the X–Y plane. These vibrational modes are influenced by the interlayer structure of NbSe_2_, and their frequencies shift due to structural changes as the number of layers decreases. The E^1^_2g_ mode, which corresponds to intralayer vibrations, experiences an upshift in frequency as interlayer interactions weaken, strengthening intralayer bonding. Conversely, the A_1g_ mode, associated with interlayer vibrations, shows a downshift as interlayer interactions diminish.

**Figure 3:**
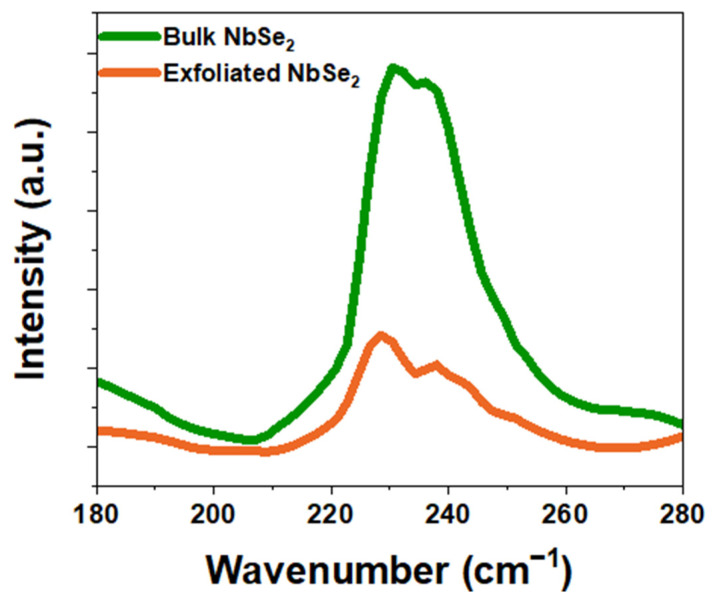
Raman spectroscopy analysis comparing Bulk NbSe₂ and Exfoliated NbSe₂, highlighting characteristic peak shifts and changes in intensity due to exfoliation.

As shown in Figure 3, the Raman peaks for bulk NbSe_2_ were observed at approximately 230.43 cm⁻^1^ for A_1g_ and 236.17 cm⁻^1^ for E^1^_2g_. For exfoliated NbSe_2_ nanosheets, the A_1g_ peak shifted downward to 228.52 cm⁻^1^, while the E^1^_2g_ peak shifted upward to 238.08 cm⁻^1^ [46,47]. These shifts confirm the structural transformation from bulk state to nanosheet form and demonstrate the success of the exfoliation process.

### 3.3. Electrical Properties of NbSe_2_ Membranes

**Figure 4:**
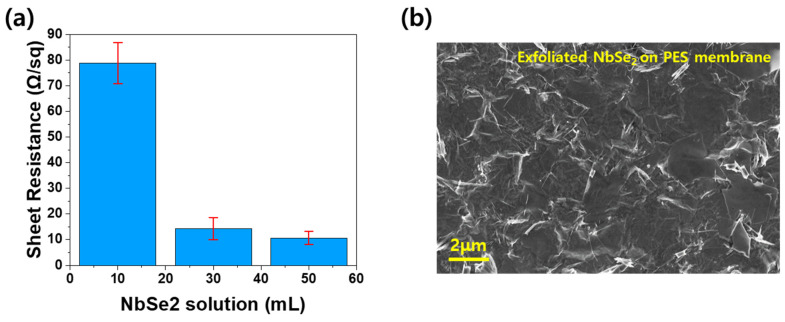
(**a**) Sheet resistance of NbSe_2_ membranes prepared with 10 mL, 30 mL, and 50 mL of solution; (**b**) SEM image of exfoliated NbSe_2_ nanosheets densely packed on a PES membrane.

Before fabricating the N-HAT, the electrical properties of the NbSe_2_ membrane were evaluated, as shown in Figure 4a. The hybrid force mechanism leverages the complementary effects of electromagnetic and electrostatic forces acting simultaneously on the diaphragm. This approach mitigates the weaknesses of each force individually, thereby enhancing the overall SPL performance in terms of decibels (dB).

Electrostatic force, in this context, is generated by Coulomb force interactions between the diaphragm and the electrode plate. These forces cause the diaphragm to vibrate as alternating charge signals induce attraction and repulsion, producing sound waves. Since the diaphragm in the electrostatic mechanism plays a critical role in storing charge and generating an electrostatic field, its electrical conductivity significantly impacts speaker performance.

The electrical conductivity of the diaphragm directly influences the stability of the electrostatic field and the signal response speed. A diaphragm with low electrical conductivity cannot evenly distribute charge across its surface, leading to non-uniform electrostatic fields [40]. Such uneven fields distort acoustic output and degrade performance. Moreover, the conductivity affects the response speed to alternating signals, especially in high-frequency ranges. A diaphragm with superior electrical conductivity enables smooth charge movement, ensuring distortion-free sound output at high frequencies.

As depicted in Figure 4a, the surface resistance of the NbSe_2_ membrane was measured based on the amount of exfoliated NbSe_2_ solution filtered. Solutions with fixed concentrations of exfoliated NbSe_2_ were prepared in three volumes: 10 mL, 30 mL, and 50 mL. Each solution was vacuum-filtered through a PES filter membrane, and the resulting NbSe_2_ membranes were dried in an oven. Surface resistance measurements were conducted using the four-point probe method, yielding average values of 78.8 Ω/sq, 14.3 Ω/sq, and 10.7 Ω/sq for the 10 mL, 30 mL, and 50 mL samples, respectively. The 50 mL sample exhibited the lowest resistance, indicating the best electrical conductivity.

As previously described, NbSe_2_ has a metallic nature with excellent electrical conductivity. For a NbSe_2_ membrane to exhibit electrical conductivity, the micro-sized nanosheets must form an interconnected network across the membrane’s entire surface. Since the concentration of NbSe_2_ nanosheets in the solutions was identical, the quantity of nanosheets deposited during filtration varied with the solution volume. A larger volume resulted in more densely packed nanosheets, creating a more robust electrical network. The SEM image in Figure 4b of the membrane produced from 50 mL of solution confirms this, showing densely packed exfoliated NbSe_2_ nanosheets covering the filter membrane entirely. Therefore, the NbSe_2_ membrane fabricated using 50 mL of solution, with its superior electrical conductivity, was selected as the diaphragm for the N-HAT.

### 3.4. SPL Performance of N-HAT

The experiments conducted thus far confirmed the successful exfoliation of NbSe_2_ and evaluated the electrical properties of the NbSe_2_ membranes under various conditions. Based on these results, the NbSe_2_ membrane was applied to a hybrid acoustic transducer system capable of sound output through hybrid force, and the N-HAT was fabricated. The system was analyzed for frequency response under electromagnetic force, electrostatic force, and hybrid force conditions.

**Figure 5:**
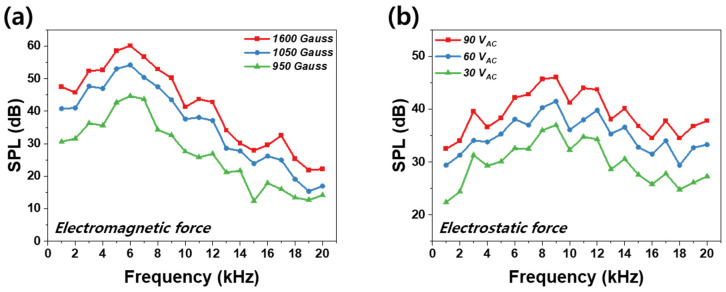
(**a**) SPL frequency response under electromagnetic force with magnetic field strengths of 950 Gauss, 1050 Gauss, and 1600 Gauss; (**b**) SPL frequency response under electrostatic force with AC voltages of 30 V, 60 V, and 90 V.

First, as shown in Figure 5a, SPL measurements were conducted for electromagnetic force, varying the strength of the magnet. The electromagnetic mechanism, commonly used in everyday devices such as speakers, headphones, and earphones, is a widely adopted approach due to its simplicity and efficiency. In the electromagnetic mechanism, alternating current corresponding to sound signals flows through a copper coil, generating a magnetic field. This magnetic field changes direction according to the AC signal and interacts with the static magnetic field of a permanent magnet, resulting in alternating attraction and repulsion forces. Consequently, the coil vibrates, causing the diaphragm to vibrate in turn, compressing and expanding air to produce sound waves.

The force generated in this process is Lorentz force, which arises from the interaction between the magnetic field of the permanent magnet and the current flowing through the solenoid-shaped coil. This can be expressed by the equation below [35].

(1) $$F_{L}=I\cdot \int_{0}^{l}Bdl,$$

In this equation, I represents the amount of current, and B denotes the magnetic flux density. As the equation indicates, the electromagnetic force is significantly influenced by both the current and the magnetic field. However, the given equation applies to a solenoid with a cylindrical hollow structure, whereas the N-HAT in this study utilizes a planar copper coil. The Lorentz force equation for this configuration is expressed as follows [48]:

(2) $$F_{L}=\sum_{n=1}^{N}I\cdot 2\pi \cdot R_{n}\cdot B_{r}(R_{n}),$$

At this point, when counting the turns of the coil outward from the center, R_n_ represents the radius of the n-th turn of the planar coil, and B_r_(R_n_) denotes the magnetic flux density at the n-th copper wire

As shown in Figure 5a, frequency response was measured using three magnets with different field strengths. As inferred from the equations above, a higher flux density of the magnetic field results in stronger Lorentz force—i.e., greater attraction and repulsion forces applied to the diaphragm—leading to improved SPL performance. Accordingly, SPL measurements were conducted on N-HAT devices equipped with magnets of 950 Gauss, 1050 Gauss, and 1600 Gauss. The results showed that the N-HAT with the 1600 Gauss magnet exhibited the highest SPL performance. However, as illustrated in the graph, all three cases showed a decline in SPL performance in the high-frequency range above 6 kHz. This decline can be attributed to two primary factors. First, in the electromagnetic mechanism, the diaphragm structure consists of a metal coil and adhesive film. Consequently, the force does not act uniformly across the diaphragm’s entire surface; instead, the vibration is primarily driven by the metallic coil. As the frequency of the alternating current applied to the copper coil increases, the impedance of the coil also rises, which in turn reduces the current [49]. Thus, structural limitations and the impedance increase at high frequencies weaken the electromagnetic force in this range [50].

Next, frequency response measurements were conducted when only electrostatic force was applied to the NbSe_2_ membrane. As shown in Figure 5b, frequency response was measured under alternating voltage levels of 30 V_AC_, 60 V_AC_, and 90 V_AC_. The results indicate that the highest SPL performance was achieved at 90 V_AC_. In the electrostatic mechanism, a conductive diaphragm is subjected to alternating current signals corresponding to sound signals, causing the diaphragm’s charge to alternate. The ITO electrode plate positioned in front of the diaphragm is charged with a constant voltage by the power supply. Attraction and repulsion forces arise due to changes in the charge direction of the diaphragm, causing it to vibrate. These forces, known as Coulomb forces, can be expressed by the following equation [39]:

(3) $$F_{c}=\frac{\varepsilon A}{2d^{2}}{\left(V_{AC}+V_{DC}\right)}^{2},$$

Here, A represents the area of the NbSe_2_ membrane, d is the distance between the electrode plate and the NbSe_2_ membrane, ε is the permittivity of the air medium, and V_AC_ and V_DC_ are the AC and DC voltages, respectively. In the N-HAT system, V_DC_, which is applied to the ITO electrode plate, is maintained at a constant 80 V, while V_AC_ is the alternating voltage applied to the NbSe_2_ membrane.

**Figure 6:**
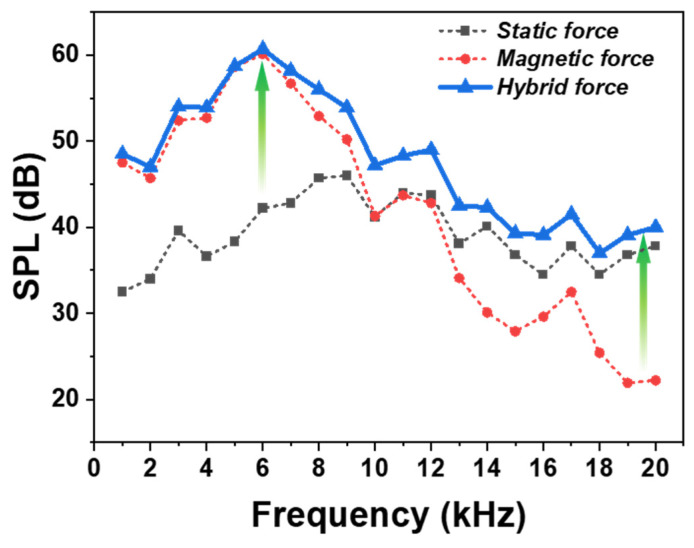
Frequency response of N-HAT showing hybrid force outperforming electromagnetic and electrostatic forces, with 44% higher SPL at 6 kHz and 80% at 20 kHz.

As seen from Equation (3), when the DC voltage on the electrode plate remains constant, a larger AC voltage applied to the diaphragm results in stronger Coulomb force. Following this principle, the NbSe_2_ membrane exhibited the best SPL performance when subjected to a 90 V_AC._ After optimizing the SPL performance for both electromagnetic and electrostatic forces through the preceding experiments, frequency response measurements were conducted for the hybrid force mechanism, where both forces act simultaneously. As shown in Figure 6, electromagnetic force dominates below 10 kHz, but its output declines at higher frequencies, where electrostatic force becomes more significant. At 6 kHz, the SPL for electrostatic force alone was 42.2 dB, while the hybrid force achieved 60.7 dB, representing a 44% improvement. Conversely, at 20 kHz, the SPL for electromagnetic force alone was 22.2 dB, whereas the hybrid force reached 40 dB, indicating an 80% performance enhancement.

As depicted in the graph, the hybrid force consistently maintained higher SPL performance across the entire frequency range compared to either electromagnetic or electrostatic force alone. The relationship between the combined forces acting on the diaphragm and the resulting vibration modes of the diaphragm can be expressed by the following equation [42]:

(4) $$F_{t}=F_{L}+F_{c}=M\frac{d^{2}z}{dt^{2}}+R\frac{dz}{dt}+\frac{1}{C}z,$$

Here, F_t_ represents the sum of the two forces, while M, R, and C denote the equivalent mass, damping, and compliance, respectively. z represents the vibration amplitude, which refers to the amplitude of vibration in the thickness direction of the diaphragm when it vibrates. As indicated in Equation (4), the combined force F_t_, resulting from the simultaneous application of two forces, is greater than the force from a single source, thereby increasing z as well.

The following equation expresses the relationship between z and the sound pressure P(d) [40]:

(5) $$P\left(d\right)=\rho {\left(2\pi f\right)}^{2}\int_{o}^{r}\frac{z(k)}{\sqrt{d^{2}+k^{2}}}kdk,$$

Here, f represents the frequency, d is the distance from the diaphragm, ρ is the air density, and z(k) is the vibration amplitude at a point located k distance from the center of the diaphragm. As indicated by this equation, an increase in z results in a higher vibration amplitude of the diaphragm, which naturally increases the sound pressure P(d) exerted on the air. Consequently, the Sound Pressure Level (SPL) also increases. The variation in SPL frequency response observed in Figure 6 is influenced by the interaction of the hybrid force mechanism, the diaphragm’s physical properties, and the system design. The hybrid mechanism, which combines electrostatic and electromagnetic forces, results in frequency-dependent resonance and non-resonance states due to displacement limitations and energy losses at different frequencies. Additionally, the lightweight and sensitive NbSe_2_-based diaphragm, along with structural damping and energy loss in the system, contributes to the observed changes, highlighting the complex interplay of these factors.

To elaborate further, the hybrid force mechanism involves the simultaneous application of electromagnetic and electrostatic forces to the diaphragm. This combined action increases the vibration amplitude of the diaphragm. As the vibration amplitude rises, the pressure exerted on the air increases, leading to an enhanced SPL output.

## 4. Conclusions

In this study, a hybrid force acoustic transducer was developed using NbSe_2_, a TMDC known for its excellent electrical conductivity and superconducting properties. NbSe_2_ was successfully exfoliated into nanosheets via lithium-ion intercalation and used as a diaphragm material, retaining its thin-layered structure and high conductivity to enhance performance. The hybrid mechanism, combining electromagnetic and electrostatic forces, addressed the limitations of each individual approach. Notably, it achieved up to 80% improved SPL performance in the high-frequency range above 6 kHz. This overcame challenges like reduced output in the high-frequency range for electromagnetic systems and low sound pressure in the low-frequency range for electrostatic systems. The NbSe_2_-based transducer demonstrated exceptional performance across a wide frequency range. These findings highlight the suitability of NbSe_2_ for acoustic applications and the potential of hybrid mechanisms in next-generation devices. Future studies could explore other TMDCs, such as MoS_2_, WS_2_, and WSe_2_, to compare performance and optimize material selection for specific applications. Understanding how TMDC properties influence SPL and frequency response would further enhance transducer design.

## Data Availability

The original contributions presented in this study are included in the article. Further inquiries can be directed to the corresponding author.
